# Two simple-to-use web-based nomograms to predict overall survival and cancer-specific survival in patients with extremity fibrosarcoma

**DOI:** 10.3389/fonc.2022.942542

**Published:** 2023-02-13

**Authors:** Yubo Li, Jianing Yang, Long Zhao, Bin Chen, Yongsheng An

**Affiliations:** Department of Orthopedics, Affiliated Hospital of Chengde Medical University, Chengde, Hebei, China

**Keywords:** fibrosarcoma, prognosis, predictive model, soft tissue sarcoma, survival

## Abstract

**Background:**

Fibrosarcoma is a rare sarcoma of the soft tissue in adults, occurring most commonly in the extremities. This study aimed to construct two web-based nomograms to predict overall survival (OS) and cancer-specific survival (CSS) in patients with extremity fibrosarcoma (EF) and validate it with multicenter data from the Asian/Chinese population.

**Method:**

Patients with EF in the Surveillance, Epidemiology, and End Results (SEER) database between 2004 and 2015 were included in this study and were randomly divided into a training cohort and a verification cohort. The nomogram was developed based on the independent prognostic factors determined by univariate and multivariate Cox proportional hazard regression analyses. The predictive accuracy of the nomogram was validated with the Harrell’s concordance index (C-index), receiver operating curve, and calibration curve. Decision curve analysis (DCA) was utilized to compare the clinical usefulness between the novel model and the existing staging system.

**Result:**

A total of 931 patients finally were obtained in our study. Multivariate Cox analysis determined five independent prognostic factors for OS and CSS, namely, age, M stage, tumor size, grade, and surgery. The nomogram and the corresponding web-based calculator were developed to predict OS (https://orthosurgery.shinyapps.io/osnomogram/) and CSS (https://orthosurgery.shinyapps.io/cssnomogram/) probability at 24, 36, and 48 months. The C-index of the nomogram was 0.784 in the training cohort and 0.825 in the verification cohort for OS and 0.798 in the training cohort and 0.813 in the verification cohort for CSS, respectively, indicating excellent predictive performance. The calibration curves showed excellent agreement between the prediction by the nomogram and actual outcomes. Additionally, the results of DCA showed that the newly proposed nomogram was significantly better than the conventional staging system with more clinical net benefits. The Kaplan–Meier survival curves showed that patients assigned into the low-risk group had a more satisfactory survival outcome than the high-risk group.

**Conclusion:**

In this study, we constructed two nomograms and web-based survival calculators including five independent prognostic factors for the survival prediction of patients with EF, which could help clinicians make personalized clinical decisions.

## Introduction

According to the World Health Organization classification of bone and soft tissue sarcoma (STS), fibrosarcoma is an infrequently encountered malignant neoplasm of mesenchymal origin and comprises approximately 5%–8% of adult STS (1, 2). From a pathological perspective, it mainly arises from transformed spindle-forming fibroblasts that had an excessive rate of division (3). Surgical excision is currently the standard treatment for fibrosarcoma; doxorubicin-based chemotherapy is considered as the main adjuvant therapy method (4). Recently, with the development and application of molecular-targeted drugs, neoadjuvant chemotherapy, and advances in limb-sparing surgery, the outcome of fibrosarcoma has improved (5), while the overall prognosis remains poor due to highly localized aggressiveness and low response rates to chemotherapy. Unlike infantile fibrosarcoma, 80% of adult-type fibrosarcomas are highly malignant, with an overall 5-year survival rate of approximately 40%–50% (6). More than half of all fibrosarcomas occur in the extremities and rank third among all of the STS of the extremities (7). It was reported that the treatment and prognoses of STS differ according to the anatomical location (e.g., head and neck, viscera, retroperitoneum, and extremity) (8). Patients with extremity fibrosarcoma (EF) and this subset represent a unique population requiring further consideration.

Nowadays, the American Joint Committee on Cancer (AJCC) tumor node metastasis TNM staging system, incorporated with the anatomical information of the extent of the primary tumor (T), regional lymph nodes (N), and distant metastases (M), is widely used to assess the prognosis of cancer patients. Although the TNM staging system is considered as standard protocol for prognosis evaluation for patients with malignancies, several studies have shown that the current TNM staging system for STS patients is inadequate in regard to the correctness of N staging and individualized variability (9, 10). In addition, the absence of a staging system with clinical and pathological features will also lead to a dilemma in treatment algorithm and prognostic assessment. Recently, the nomogram, an easy-to-understand multivariate visualization model, has been widely used for personalized prediction of patients with a variety of malignancies (11–13). Ye et al. developed a nomogram for predicting overall survival (OS) in patients with liposarcoma of the extremities by integrating six clinical variables, which exhibited higher discrimination than each single predictor (14). Furthermore, a prediction model proposed by Song and colleagues was shown to have higher stability and accuracy than the conventional TNM staging system and could be considered as a good alternative to the system (15). Although some predictive models for patients with fibrosarcoma have been constructed previously, the difference in accuracy between relying on these models and relying on the TNM staging system for prognostic assessment has not been investigated in detail. Therefore, it seems helpful to develop a novel prognostic model with excellent predictive performance for special populations and to incorporate it into daily clinical practice. Consequently, using the population-based data from the Surveillance, Epidemiology, and End Results (SEER) database, this study aims to develop two prognostic nomograms and web-based survival calculators for predicting the survival rate of patients with EF.

## Methods

### Study population

**Figure 1:**
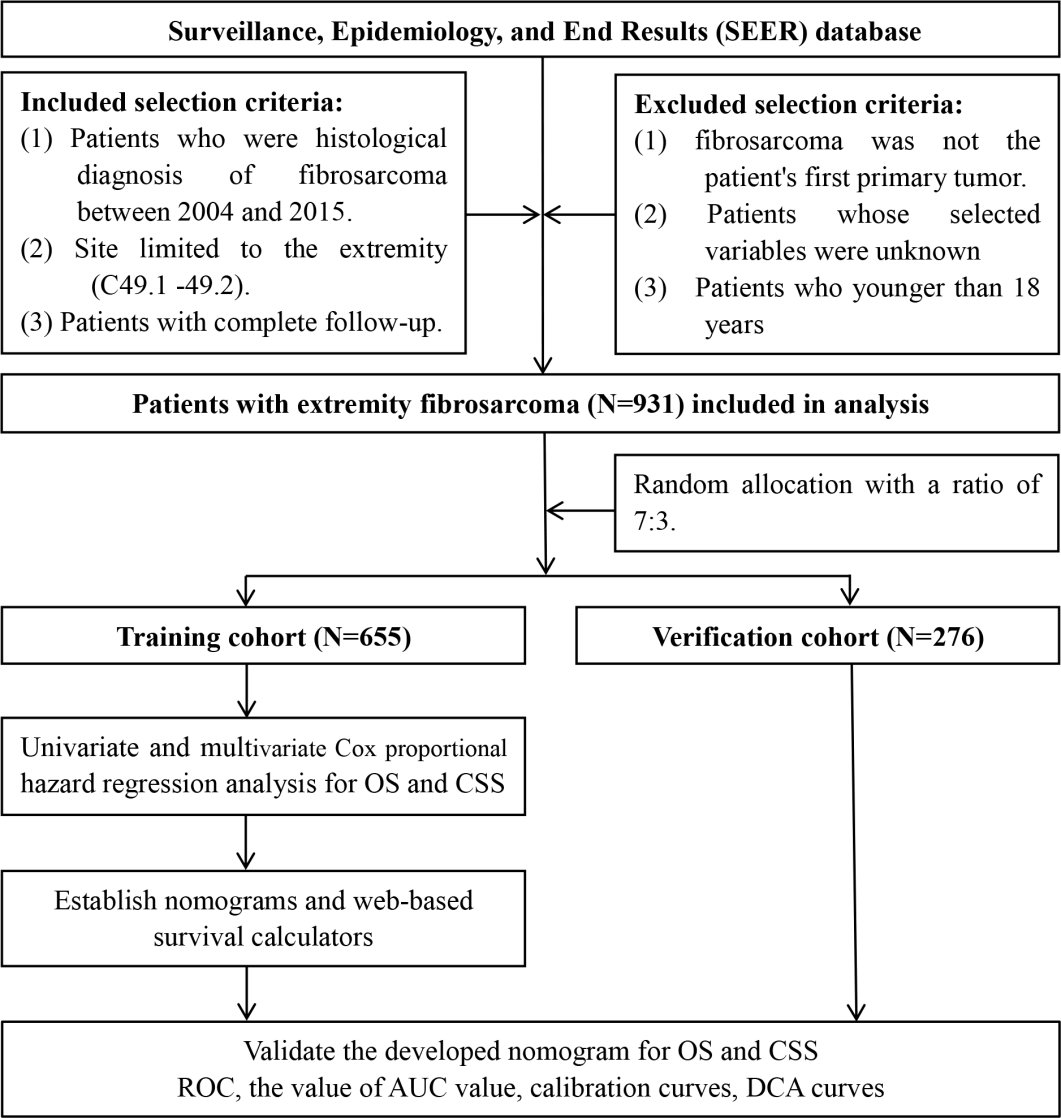
Flow diagram of selecting patient records from the Surveillance, Epidemiology, and End Results (SEER) database.

The data of patients with EF were downloaded from the SEER database by the SEER*Stat software version 8.4.0 during the period from 2004 to 2015. As shown in **Figure 1**, we identified eligible cases if sufficient data were available from the database. The inclusion criteria of patient selection were as follows: (a) patients whose diagnosis of fibrosarcoma (ICD-O-3 Hist/behave: 8810-8814, 8823, 8832, 8833, 9321, and 9330) was confirmed by histology during 2004–2015; (b) site limited to the extremity (Primary Site-labeled: C49.1-49.2); and (c) patients with complete follow-up. The exclusion criteria were as follows: (a) patients whose selected variables were unknown; (b) fibrosarcoma was not the patient’s first primary tumor; (c) patients diagnosed with autopsies or death certificates; and (d) patients who are younger than 18 years. Selected variables included age, sex, race, primary site, tumor size, grade, T stage, N stage, M stage, surgery, radiotherapy, and chemotherapy. In these variables, the grade was classified into well differentiation (I–II) and poor differentiation (III–IV). X-tile software provides the best cut-off point and changes continuous variables into categorical variables; therefore, the age at diagnosis is categorized as younger than 67, 67–79 years, and older than 79 years; the tumor size (CS tumor size, 2004–2015) of patients is divided into three groups, namely, <65 mm, 65–99 mm and >99 mm. TNM staging was recorded for all patients based on the AJCC TNM classification, 7th edition. It is worth mentioning that we have classified the type of surgery for patients undergoing surgery into three categories, including partial resection, radical resection, and amputation, according to the surgical modality codes provided in the database. The endpoint of this study was determined to be OS and cancer-specific survival (CSS); OS was defined as the time between the date of the disease diagnosis and the date of death from any disease cause, and CSS was defined as the time from the diagnosis to death from EF. **Figure 1** shows the workflow for the study.

### Statistical analysis

In our study, the statistical method was performed in Statistical Product and Service Solutions 26.0 and R software (version 4.1.1). Differences in baseline characteristics were examined using the chi-square test. The total cohort of patients with EF was randomized into two cohorts by a 7:3 ratio, including the training cohort (n = 655) and verification cohort (n=276). Univariate Cox regression analysis was firstly used to explore OS-related variables. Variables with P < 0.05 were further analyzed by multivariate analysis. Then, the variables with P<0.05 in multivariate Cox analysis were finally determined as the independent prognostic factors of patients with EF. Hazard ratios (HRs) and 95% confidence intervals (CIs) were also calculated to show the relevance between prognostic factors and survival. Afterward, we developed two prognostic nomograms to predict OS and CSS at the time of 24, 36, and 48 months, respectively; meanwhile, two web-based survival rate calculators were further established based on the nomograms using the “Dynnom” package. A k-fold (k = 10) cross-validation method was performed to validate the newly proposed model. The time-dependent receiver operating characteristic (ROC) curves were plotted, and the value of the area under the curve (AUC) was used to estimate the discrimination of the nomogram. The calibration curves were also generated. Moreover, the time-dependent ROC curve of identified independent prognostic variables was established to compare the AUC between each single factor and the comprehensive model; decision curve analysis (DCA) was utilized to assess the clinical usefulness of the nomogram and to compare the predictive accuracy between the AJCC TNM staging system and the developed nomogram. Moreover, we calculated the total point of all patient-based nomograms and divided it into three subgroups according to the optimal cut-off value determined by X-tile, namely, high-risk, medium-risk, and low-risk groups. The Kaplan–Meier survival analysis with log-rank tests was conducted to compare the survival difference among the three groups. P < 0.05 was considered statistically significant.

## Results

### Baseline data of patients with extremity fibrosarcoma

In this study, we finally included 931 patients with EF. The study population was randomly allocated into two cohorts, including the training cohort (n = 655) and verification cohort (n = 276). Overall, most patients were white (79.16%, 737 cases). Patients in the <67 years (64.34%, 599 cases) age group made up the majority of the study sample. The men-to-women ratio was close to 1:1. The most common primary site of patients with EF was the lower limb, making up 68.10% (634 cases), while the upper limb made up 31.90% (297 cases). According to the AJCC TNM staging system, 426 cases (45.76%) were staged as T1, while 505 cases (54.24%) were staged as T2. In terms of the N and M stages, 921 cases (98.93%) were staged as N0, 10 cases (1.07%) as N1, 902 cases (96.89%) were staged as M0, and another 29 (3.11%) cases were staged as M1. Furthermore, there is basically a 50–50 split between well differentiation (50.91%) and poor differentiation (49.09%) tumors. In terms of treatment information, only half of the patients (53.49%) undergoing radiotherapy and other cases were not treated. While most patients received the surgery (913 cases, 98.07%), of these, the most commonly accepted surgical procedure is radical resection (544 cases, 58.43%). The detailed demographic and clinicopathological information of the two cohorts is listed in **Table 1**.

**Table 1:** The demographic and clinicopathologic information of patients with extremity fibrosarcoma (EF).

Variables		Total cohort (n, %)	Training cohort (n, %)	Verification cohort (n, %)	p-value
		N = 931	N = 655	N = 276	
**Age**	<67 years,	599 (64.34)	419 (63.97)	180 (65.22)	0.779
	67–79 years	209 (22.45)	151 (23.05)	58 (21.01)	
	>79 years	123 (13.21)	85 (12.98)	38 (13.77)	
**Race**	Black	100 (10.74)	67 (10.23)	33 (11.96)	0.619
	Other	94 (10.10)	69 (10.53)	25 (9.06)	
	White	737 (79.16)	519 (79.24)	218 (78.99)	
**Sex**	Female	449 (48.23)	318 (48.55)	131 (47.46)	0.817
	Male	482 (51.77)	337 (51.45)	145 (52.54)	
**Primary site**	Lower limb	634 (68.10)	447 (68.24)	187 (67.75)	0.944
	Upper limb	297 (31.90)	208 (31.76)	89 (32.25)	
**Grade**	Grade I–II	474 (50.91)	326 (49.77)	148 (53.62)	0.316
	Grade III–IV	457 (49.09)	329 (50.23)	128 (46.38)	
**T stage**	T1	426 (45.76)	306 (46.72)	120 (43.48)	0.404
	T2	505 (54.24)	349 (53.28)	156 (56.52)	
**N stage**	N0	921 (98.93)	648 (98.93)	273 (98.91)	1.000
	N1	10 (1.07)	7 (1.07)	3 (1.09)	
**M stage**	M0	902 (96.89)	638 (97.40)	264 (95.65)	0.2305
	M1	29 (3.11)	17 (2.60)	12 (4.35)	
**Surgery**	No	18 (1.93)	15 (2.29)	3 (1.09)	0.3645
	Partial resection	341 (36.63)	247 (37.71)	94 (34.06)	
	Radical resection	544 (58.43)	375 (57.25)	169 (61.23)	
	Amputation	28 (3.01)	18 (2.75)	10 (3.62)	
**Radiotherapy**	No	433 (46.51)	296 (45.19)	137 (49.64)	0.2418
	Yes	498 (53.49)	359 (54.81)	139 (50.36)	
**Chemotherapy**	No/Unknown	834 (89.58)	588 (89.77)	246 (89.13)	0.8613
	Yes	97 (10.42)	67 (10.23)	30 (10.87)	
**Tumor size**	<65 mm	545 (58.54)	390 (59.54)	155 (56.16)	0.2128
	65–99 mm	187 (20.09)	135 (20.61)	52 (18.84)	
	>99 mm	199 (21.37)	130 (19.85)	69 (25.00)	

### Independent predictors for overall survival and cancer-specific survival of patients with extremity fibrosarcoma

In the univariate regression analysis of OS and CSS, we found that age, T stage, N stage, M stage, grade, tumor size, chemotherapy, and surgery were all significant influencing factors (p < 0.05). All these variables were then incorporated into the multivariate Cox analysis; age, grade, M stage, tumor size and surgery were finally identified as the independent prognostic factors for OS and CSS (**Tables 2**, **3**).

**Table 2:** Univariate and multivariate Cox analyses of overall survival for patients with EF.

Variables	Univariate analysis		Multivariate analysis	
	HR (95%CI)	p-value	HR (95%CI)	p-value
Age				
<67 years	Reference		Reference	
67–79 years	2.38 (1.61–3.53)	<0.001	2.45 (1.63–3.68)	<0.001
>79 years	7.53 (5.19–10.93)	<0.001	7.73 (5.1–11.72)	<0.001
Sex				
Female	Reference			
Male	1.08 (0.79–1.48)	0.6190		
Race				
Black	Reference			
Other	1.11 (0.54–2.28)	0.7730		
White	1.12 (0.65–1.95)	0.6810		
Primary site				
Lower limb	Reference			
Upper limb	1 (0.71–1.39)	0.9870		
T stage				
T1	Reference		Reference	
T2	1.68 (1.21–2.32)	0.0020	0.77 (0.43–1.38)	0.3813
N stage				
N0	Reference		Reference	
N1	11.55 (5.37–24.85)	<0.001	1.41 (0.52–3.81)	0.4927
M stage				
M0	Reference		Reference	
M1	5.98 (3.38–10.56)	<0.001	3.92 (1.87–8.19)	<0.001
Tumor size				
<65 mm	Reference		Reference	
65–99 mm	1.23 (0.8–1.88)	0.3460	1.27 (0.67–2.38)	0.4626
>99 mm	3.03 (2.14–4.29)	<0.001	3.12 (1.74–5.6)	<0.001
Grade				
Well differentiation	Reference		Reference	
Poor differentiation	2.28 (1.63–3.18)	<0.001	1.51 (1.06–2.15)	0.0212
Chemotherapy				
No/Unknown	Reference		Reference	
Yes	1.77 (1.15–2.72)	0.0090	1.55 (0.95–2.55)	0.0812
Radiotherapy				
No	Reference			
Yes	0.98 (0.72–1.34)	0.8870		
Surgery				
No	Reference		Reference	
Partial resection	0.23 (0.11–0.47)	<0.001	0.21 (0.1–0.43)	<0.001
Radical resection	0.2 (0.1–0.41)	<0.001	0.19 (0.09–0.39)	<0.001
Amputation	0.42 (0.16–1.14)	0.0880	0.4 (0.15–1.08)	0.0695

**Table 3:** Univariate and multivariate Cox analyses of cancer-specific survival for patients with EF.

Variables	Univariate analysis		Multivariate analysis	
	HR (95%CI)	p-value	HR (95%CI)	p-value
Age				
<67 years	Reference		Reference	
67–79 years	1.3 (0.77–2.2)	0.3250	1.41 (0.81–2.45)	0.2232
>79 years	3.27 (1.94–5.51)	<0.001	3.18 (1.73–5.84)	<0.001
Sex				
Female	Reference			
Male	1.54 (1–2.37)	0.0500		
Race				
Black	Reference			
Other	1.19 (0.49–2.88)	0.6930		
White	0.91 (0.46–1.83)	0.7990		
Primary site				
Lower limb	Reference			
Upper limb	0.86 (0.54–1.36)	0.5140		
T stage				
T1	Reference		Reference	
T2	2.54 (1.58–4.09)	<0.001	0.73 (0.29–1.8)	0.4881
N stage				
N0	Reference		Reference	
N1	21.2 (9.67–46.48)	<0.001	2.27 (0.77–6.72)	0.1391
M stage				
M0	Reference		Reference	
M1	11.74 (6.49–21.22)	<0.001	4.22 (1.88–9.47)	<0.001
Tumor size				
<65 mm	Reference		Reference	
65–99 mm	1.41 (0.74–2.66)	0.2940	1.45 (0.55–3.85)	0.4508
>99 mm	5.6 (3.5–8.95)	<0.001	4.96 (2.06–11.99)	<0.001
Grade				
Well differentiation	Reference		Reference	
Poor differentiation	3.86 (2.32–6.42)	<0.001	2.42 (1.42–4.14)	0.0012
Chemotherapy				
No	Reference		Reference	
Yes	3.41 (2.12–5.5)	<0.001	1.77 (0.98–3.17)	0.0563
Radiotherapy				
No	Reference			
Yes	1.49 (0.96–2.33)	0.0750		
Surgery				
No	Reference		Reference	
Partial resection	0.16 (0.06–0.43)	<0.001	0.18 (0.07–0.48)	<0.001
Radical resection	0.25 (0.1–0.62)	0.0030	0.21 (0.08–0.55)	0.0016
Amputation	0.44 (0.12–1.65)	0.2240	0.39 (0.1–1.5)	0.1697

### Construction and validation of the nomogram

**Figure 2:**
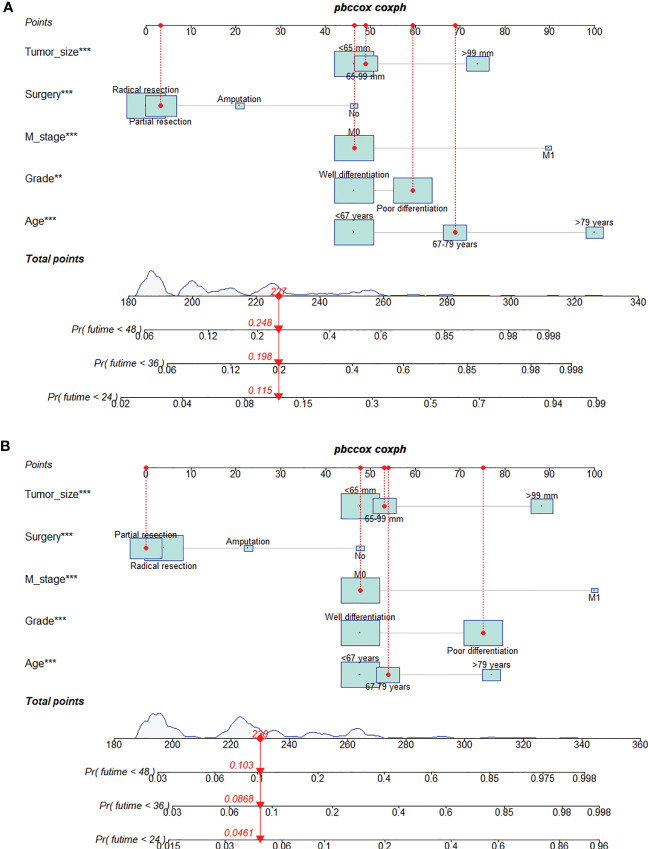
Prognostic nomogram for predicting the 24-, 36-, and 48-month overall survival (OS) **(A)** and cancer-specific survival (CSS) **(B)** of patients with extremity fibrosarcoma (EF). The meaning of the symbol **, *** represented the statistical differentiation of the variable.

**Figure 3:**
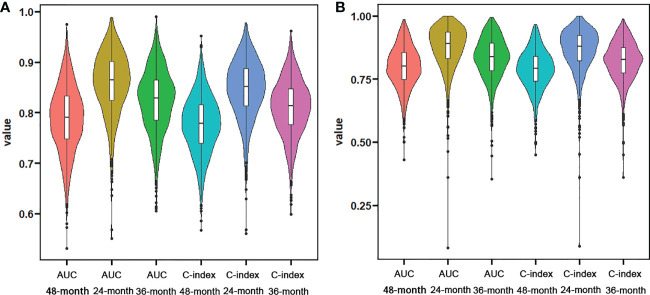
Violin plots showing the result of k-fold (k=10) cross-validation for OS analysis **(A)** and CSS analysis **(B)**.

**Figure 4:**
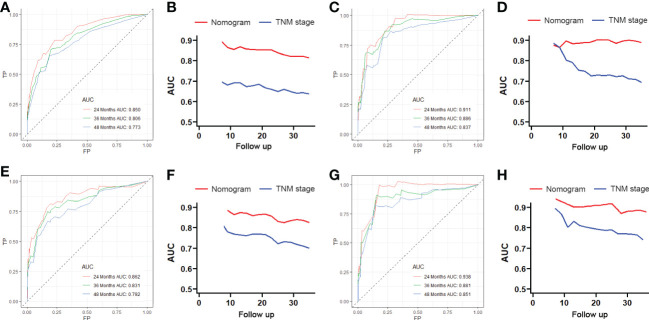
The receiving operator characteristic (ROC) curves for 24-, 36-, and 48-month OS in the training cohort **(A)** and verification cohort **(B)**. Comparison of time-dependent ROC curves between the American Joint Committee on Cancer (AJCC) TNM staging system and the nomogram in the training cohort **(C)** and verification cohort **(D)**. The ROC curves for 24-, 36-, and 48-month CSS in the training cohort **(E)** and verification cohort **(F)**. Comparison of time-dependent ROC curves between the AJCC TNM staging system and the nomogram in the training cohort **(G)** and verification cohort **(H)**.

**Figure 5:**
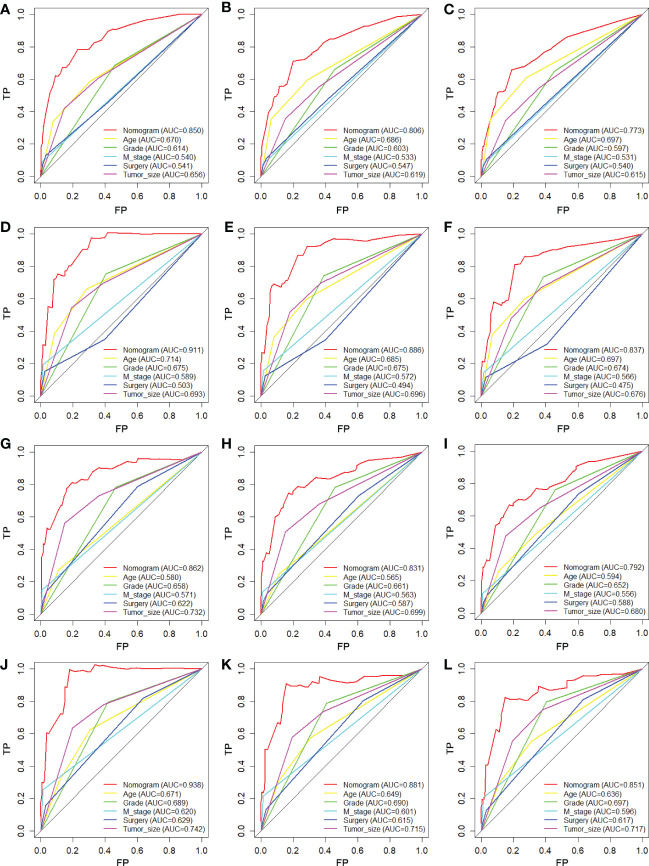
Comparison of ROC curves between the nomogram and independent predictors at 24 **(A)**, 36 **(B)**, and 48 months **(C)** in the training cohort and at 24 **(D)**, 36 **(E)**, and 48 months **(F)** in the verification cohort for OS analysis and at 24 **(G)**, 36 **(H)**, and 48 months **(I)** in the training cohort and at 24 **(J)**, 36 **(K)**, and 48 months **(L)** in the verification cohort for CSS analysis.

**Figure 6:**
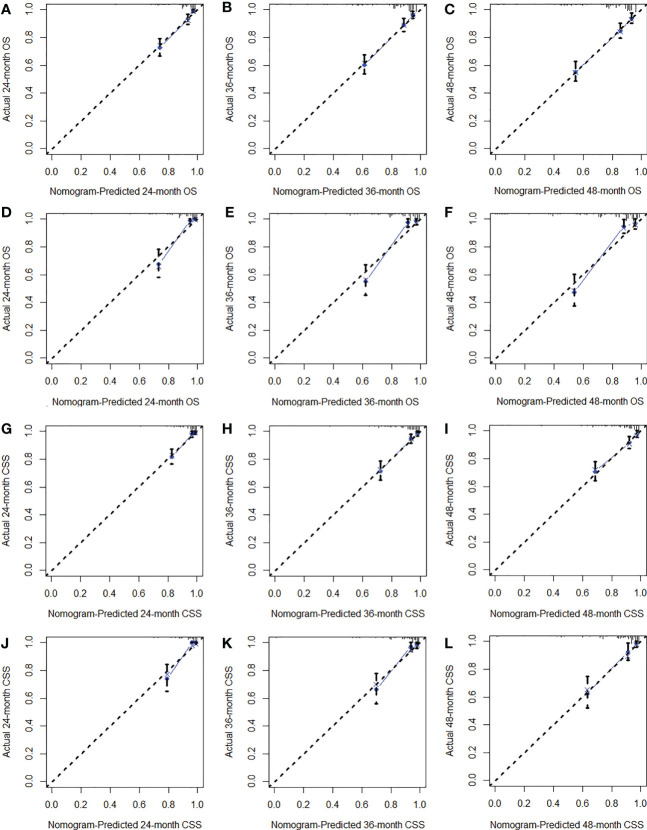
The calibration curves of the training cohort **(A–C)** and verification cohort **(D–F)** for OS analysis and of the training cohort **(G–I)** and verification cohort **(J–L)** for CSS analysis.

**Figure 7:**
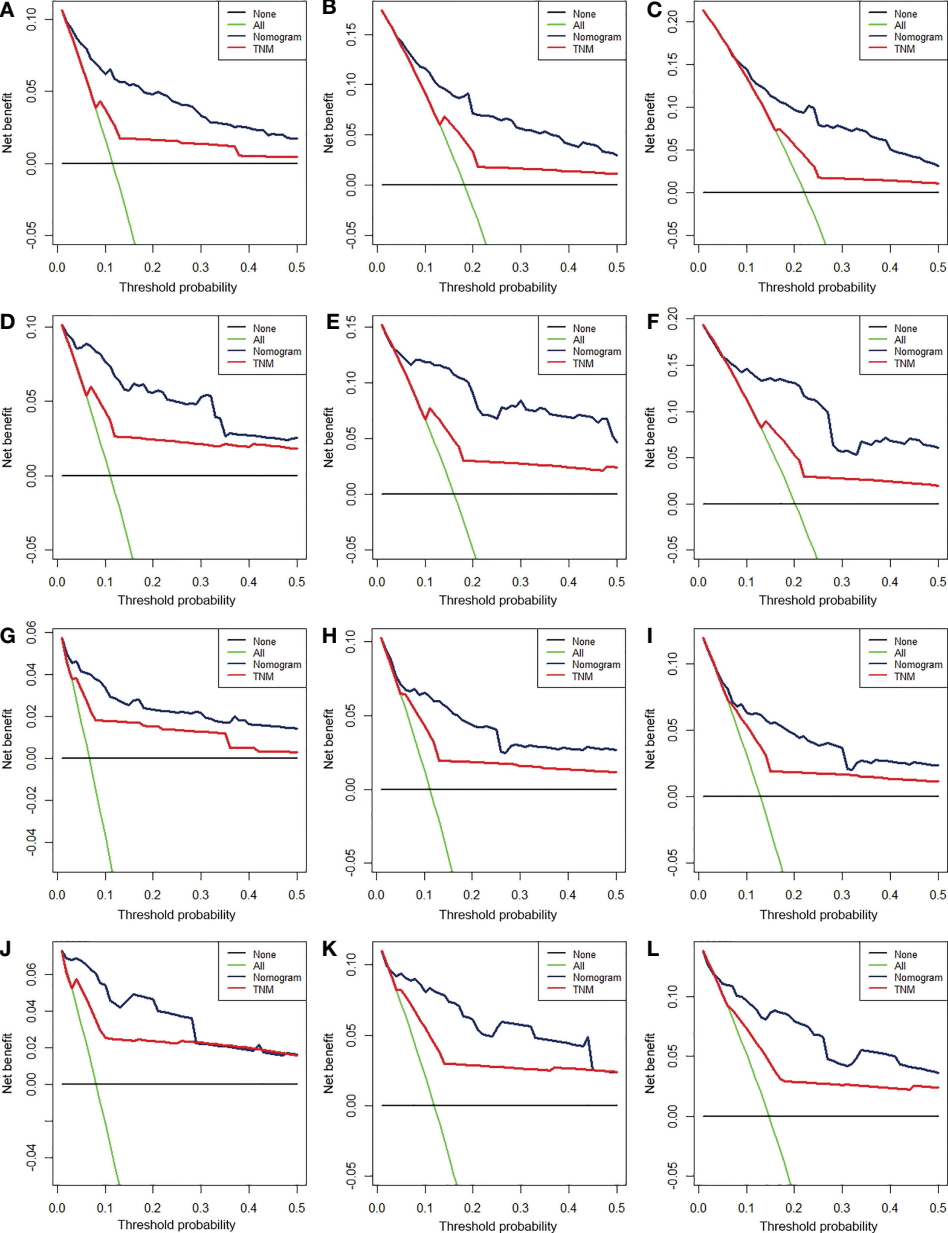
The decision curve analysis (DCA) of 24- **(A)**, 36- **(B)**, and 48-month **(C)** OS in the training cohort and 24- **(D)**, 36- **(E)**, and 48-month **(F)** OS in the verification cohort and of 24- **(G)**, 36- **(H)**, and 48-month **(I)** CSS in the training cohort and 24- **(J)**, 36- **(K)**, and 48-month **(L)** CSS in the verification cohort.

We constructed two nomograms to predict the 24-, 36-, and 48-month OS and CSS of patients with EF based on the said factors (**Figure 2**). Based on this model, we could select the subcategories of each predictor according to individual characteristics and get specific points by drawing a vertical line to the point axis at the upper end. Then, the total points could be calculated by adding up the points of all variables together to estimate the 24-, 36-, and 48-month survival probability. These nomograms were validated in the training cohort and verification cohort, respectively. The concordance index (C-index) of the nomogram for predicting OS was 0.784 (95%CI: 0.710–0.858) in the training cohort and 0.830 (95%CI: 0.700–0.930) 0.825 (95%CI: 0.721–0.929) in the verification cohort and for predicting CSS was 0.798 (95%CI: 0.690–0.906) in the training cohort and 0.813 (95%CI: 0.731–0.895) in the verification cohort. The result of k-fold cross-validation (k=10) indicated that the values of AUC for 24, 36, and 48 months were 0.865, 0.829, and 0.791 for OS analysis and 0.889, 0.838, and 0.799 for CSS analysis (**Figure 3**). In the training cohort, the AUC values of the nomogram of OS at the time of 24, 36, and 48 months were 0.850, 0.8064, and 0.773, respectively. In the verification cohort, the AUC values of the nomogram at the time of 24, 36, and 48 months were 0.911, 0.886, and 0.837, respectively (**Figures 4A, C**). As for the CSS nomogram, the AUC values of the nomogram at the time of 24, 36, and 48 months were 0.862, 0.831, and 0.792 in the training cohort and 0.938, 0.881, and 0.851 in the verification cohort (**Figures 4E, G**). The time-dependent ROC curves in two cohorts both showed that the discrimination ability of the nomogram was better than the AJCC TNM staging system (**Figures 4B, D, F, H**). We further compared the differences of the predictive performance between each independent predictor and the comprehensive model and found that the AUC of the nomogram was higher than the AUCs of all independent predictors at different time points in both the OS nomogram and the CSS nomogram, which indicated that the nomogram had more robust discrimination than single factors in survival prediction (**Figure 5**). The calibration curves showed an optimal agreement between 24-, 36-, and 48-month prediction by nomogram and actual observation in two cohorts (**Figure 6**). As shown in **Figure 7**, the nomogram showed great positive net benefits across wide ranges of death risk in both cohorts, indicating its more favorable clinical utility in predicting 24-, 36- and 48-month OS and CSS than the TNM staging system.

**Figure 8:**
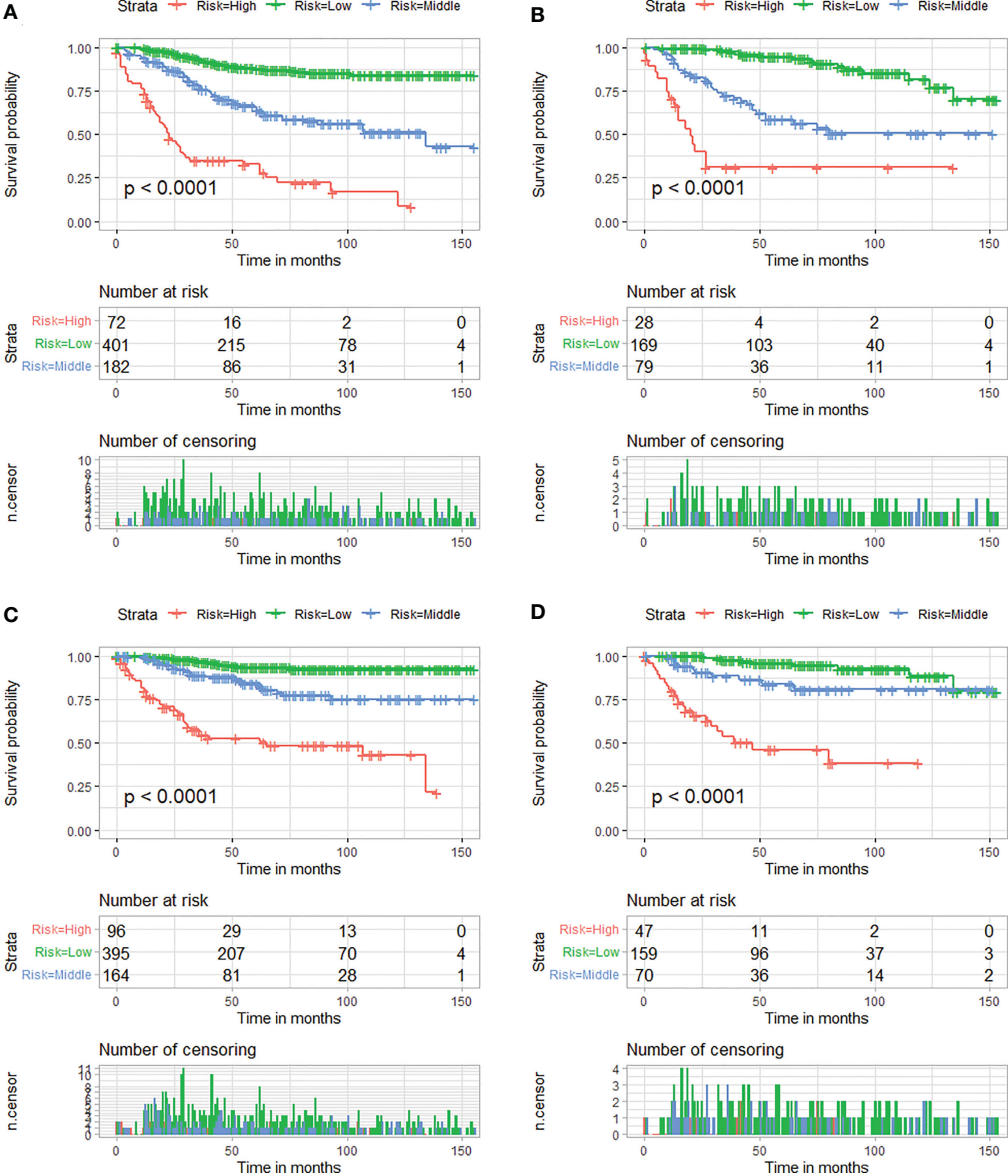
Kaplan–Meier survival curves of three mortality risk subgroups for OS in the training cohort **(A)** and verification cohort **(B)**. Kaplan–Meier survival curves of three mortality risk subgroups for CSS in the training cohort **(C)** and verification cohort **(D)**.

Moreover, according to cut-off values determined by X-tile software, in the OS analysis, patients in two cohorts were both classified into low-risk (score <228), medium-risk (score 228–266), or high-risk groups (score >266), and, in the CSS analysis, patients were both classified into low-risk (score <228), medium-risk (score 228–261), or high-risk groups (score >261). Kaplan–Meier curves showed that patients who were assigned to the high-risk group had the worst survival outcome in both cohorts (P < 0.05) (**Figure 8**).

### Development of dynamic web-based calculators for these nomograms

**Figure 9:**
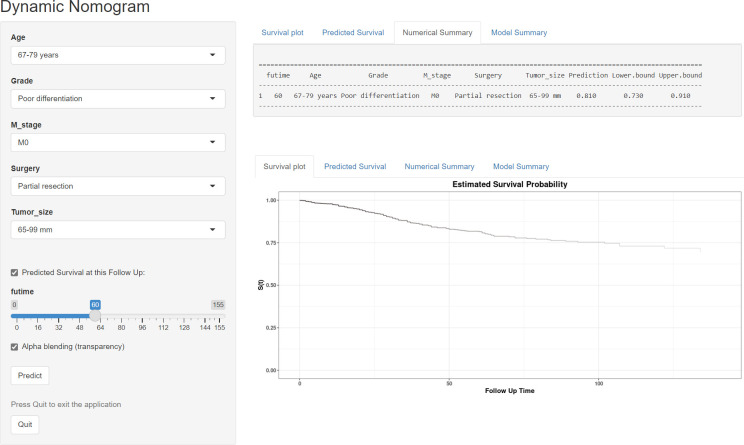
Operation interface of the web-based calculator after inputting a patient’s age, M stage, grade, surgery, and tumor size on the web and ascertaining the time point of the survival rate.

Based on the model, we developed two dynamic web-based calculators to simplify the application of these nomograms, which can be accessible *via* https://orthosurgery.shinyapps.io/osnomogram/ and https://orthosurgery.shinyapps.io/cssnomogram/ (**Figure 9**). Using the online calculator, we can conveniently obtain survival probability and its 95% CI of patients by inputting their clinical feature. For instance, for a 73-year-old patient with grade IV and a tumor size of 70 mm, M0, after receiving partial resection, the 60-month OS rate was approximately 81.0% (95% CI, 73.0%–91.0%).

## Discussion

Fibrosarcoma is a rare STS of mesenchymal cell origin arising from pathologically transformed spindle-shaped fibroblasts (16). Adult-type fibrosarcoma most often involves the deep soft tissues of the extremities, especially the thighs, knees, and arms (17). The presenting features and survival of patients with sarcoma vary depending on the involved anatomic site, which warrants a focused assessment of specific anatomic areas (18–20). Currently, the TNM staging system remains the most widely accepted system for predicting the prognosis, while the significant variability observed across more than 100 distinct STS histologic subtypes and the ignorance of the primary anatomical site of sarcoma made conventional TNM classification challenging and weakened the applicability of the AJCC staging system for the prognostic assessment of STS (21). From another point of view, the other limitations of this clinical staging system are evident as it incorporates a limited number of clinical parameters to measure the overall prognosis of patients with fibrosarcoma. Indeed, the survival outcome is reflected not only in TNM staging in the conventional AJCC staging but also in a number of other significant prognostic factors. A nomogram based on AJCC staging in combination with other important clinicopathological variables and treatment information has been widely applied as a convenient and effective tool to quantitatively predict survival time, and its accuracy and reliability have been validated in multiple cancers (22–24). Therefore, a site-specific nomogram can improve the accuracy and practical value of the prediction model.

To the best of our knowledge, several studies have developed some prognostic models focusing either on elderly patients with fibrosarcoma or on patients with fibrosarcoma who underwent surgery (25, 26); however, they included the fibrosarcoma of all anatomical sites. In addition, they did not explore in depth whether there is a difference between relying on their models and relying on the traditional TNM staging in the prognostic assessment of patients with fibrosarcoma. We believe that when a new model or calculation method is proposed, it is not enough to justify the rationality and accuracy of the newly proposed model. It significantly needs to be brought up against the existing prognostic evaluation system and draw conclusions, and such a study design can be observed in many other prognostic studies of malignancies (22, 23).

Here, our study retrospectively analyzed the data of 931 patients with EF from the SEER database and determined eight independent prognostic factors, including age, M stage, tumor size, grade, and surgery. Based on these predictors, we constructed two nomograms to predict 24-, 36-, and 48-month OS and CSS for patients with EF. The C-index, ROC curves, and calibration plots showed a good calibration ability of the nomogram, respectively. More importantly, the time-dependent ROC curves demonstrated that the newly proposed nomogram always had superior discrimination ability than the TNM staging system. In addition, DCA results indicated that the prediction of the survival rate according to the nomogram led to more net benefit than based on the TNM staging system (27). Additionally, for easier application of the model, we further developed two corresponding web-based survival calculators, from which a patient’s survival probability with 95% CI at a specific time can be reported by inputting the values of the eight variables and time.

In the present study, the survival rate was significantly poorer in patients aged 67–79 and >79 years, respectively, than in patients aged <67 years (OS analysis: 67–79 years versus <67 years: HR = 2.75, 95% CI =1.63–3.68; >79 years versus <67 years: HR = 7.73, 95% CI = 5.10–11.72, CSS analysis: 67–79 years versus <67 years: HR =1.14, 95% CI = 0.81–2.45; >79 years versus <67 years: HR = 3.18, 95% CI =1.73–5.84). This finding was in line with previous studies (28, 29).This is not surprising since older patients are often accompanied by a reduction in physiological reserve and some underlying diseases, such as diabetes, arteriosclerosis, and hypertension; these may aggravate postoperative complications (30, 31). In addition, Biau and colleagues reported that older patients were related to a higher risk of positive surgical margins (32). The said potential reasons reasonably explain the unfavorable survival outcome in elderly patients with EF.

Furthermore, the biological characteristics of the tumor were also correlated with the prognosis of cancer patients; in our study, the pathologic grade and tumor size were identified as independent predictors for the OS and CSS rates of patients with EF. A reasonable explanation was that poorer differentiation meant greater local aggressiveness, and the larger size of the primary tumor also made the goal of complete surgical excision with a negative resection margin more difficult, all of which contribute to an increased risk of local recurrence. Fibrosarcoma was reported to have a strong propensity to metastasize, and the most common metastasis site is the lung (33). Our results showed that the M stage was determined as an independent prognostic factor in patients with EF. When patients presented distant metastasis at the time of the initial diagnosis, the reasons for the inferior prognosis included not only the tumor characteristics at the primary site but also the widespread presence of circulating tumor cells. This was because the development of distant metastases required tumor cells to degrade the basement membrane and subsequently enter the systemic circulation *via* the tumor vasculature (34, 35).

Similar to other types of STSs, radical surgical excision remains the preferred treatment modality in the management of fibrosarcoma (33). This study confirmed that surgery was an independent protective factor in patients with EF (36). Moreover, our results further revealed that patients who underwent radical resection enjoyed the most favorable prognosis, followed by partial resection. Briefly, patients with EF receiving limb salvage surgery (including partial resection and radical resection) have superior clinical outcomes than those who underwent amputation. Previously, the difference of the impact of different surgical approaches to STS has been investigated; some studies from small samples or a single-center clinical cohort have shown that limb salvage surgery had a better prognosis than amputation among patients with extremity bone and STSs (37, 38). Furthermore, several other studies found that the patients receiving amputation had a higher risk of suicide and accidental death than those receiving limb salvage surgery, which might be explained by the higher degree of depression and demoralization caused by the altered gait, function, stability, strength, and appearance resulting from amputation (39, 40). These were also consistent with our observation. Nevertheless, in our study, radiotherapy and chemotherapy did not show significant prognostic significance for patients with EF. This may be due to the fact that fibrosarcoma exhibits resistance to apoptosis-inducing chemotherapeutic drugs. It has been found that the combination of recombinant TIMP-1-GPI could improve the prognosis of fibrosarcoma patients by inhibiting the growth of fibrosarcoma as well as effectively increasing tumor sensitivity to doxorubicin (41), suggesting a future interest in optimizing the clinical management of fibrosarcoma patients by modulating the tumor microenvironment and thus enhancing the chemosensitivity of the tumor.

However, there still have been several limitations. Firstly, it was difficult to avoid selective bias because this study was designed in a retrospective way. Second, there is a lack of external data from different regions due to the rarity of fibrosarcoma; therefore, further validation with data is needed to verify whether these results are generally applicable. Thirdly, we were unable to consider other factors not collected in the database that may have affected the outcomes, such as target therapy, postoperative complications, gene expression, and chromosomal alteration. Moreover, the classification of patients with unknown chemotherapy status and those who did not receive treatment into the same group is also one of the inherent flaws of the SEER database.

## Conclusions

Our study established two novel nomograms integrating determined prognostic factors and web-based survival calculators based on the nomogram to distinguish the high-risk patients with EF, which might help clinicians to develop better clinical management and treatment strategies.

## Data availability statement

The raw data supporting the conclusions of this article will be made available by the authors, without undue reservation.

## Ethics statement

Ethical review and approval was not required for the study of human participants in accordance with the local legislation and institutional requirements.

## Author contributions

YL and YA conceived and designed the study. YL and JY collected the clinical data and literature review. YL conducted the statistical analysis. LZ, JY, and BC generated the figures and tables. YL wrote the manuscript. YL and YA revised the manuscript. YA supervised the research. All authors critically read the manuscript to improve intellectual content. All authors contributed to the article and approved the submitted version.
